# Usability, Usefulness, and Acceptance of a Novel, Portable Rehabilitation System (mRehab) Using Smartphone and 3D Printing Technology: Mixed Methods Study

**DOI:** 10.2196/21312

**Published:** 2021-03-22

**Authors:** Sutanuka Bhattacharjya, Lora Anne Cavuoto, Brandon Reilly, Wenyao Xu, Heamchand Subryan, Jeanne Langan

**Affiliations:** 1 Department of Occupational Therapy Byrdine F Lewis College of Nursing and Health Professions Georgia State University Atlanta, GA United States; 2 Rehabilitation Science University at Buffalo Buffalo, NY United States

**Keywords:** stroke, rehabilitation, smart technology, 3-dimensional printing, usability

## Abstract

**Background:**

Smart technology use in rehabilitation is growing and can be used remotely to assist clients in self-monitoring their performance. With written home exercise programs being the commonly prescribed form of rehabilitation after discharge, mobile health technology coupled with task-oriented programs can enhance self-management of upper extremity training. In the current study, a rehabilitation system, namely mRehab, was designed that included a smartphone app and 3D-printed household items such as mug, bowl, key, and doorknob embedded with a smartphone. The app interface allowed the user to select rehabilitation activities and receive feedback on the number of activity repetitions completed, time to complete each activity, and quality of movement.

**Objective:**

This study aimed to assess the usability, perceived usefulness, and acceptance of the mRehab system by individuals with stroke and identify the challenges experienced by them when using the system remotely in a home-based setting.

**Methods:**

A mixed-methods approach was used with 11 individuals with chronic stroke. Following training, individuals with stroke used the mRehab system for 6 weeks at home. Each participant completed surveys and engaged in a semistructured interview. Participants’ qualitative reports regarding the usability of mRehab were integrated with their survey reports and quantitative performance data.

**Results:**

Of the 11 participants, 10 rated the mRehab system between the 67.5th and 97.5th percentile on the System Usability Scale, indicating their satisfaction with the usability of the system. Participants also provided high ratings of perceived usefulness (mean 5.8, SD 0.9) and perceived ease of use (mean 5.3, SD 1.5) on a 7-point scale based on the Technology Acceptance Model. Common themes reported by participants showed a positive response to mRehab with some suggestions for improvements. Participants reported an interest in activities they perceived to be adequately challenging. Some participants indicated a need for customizing the feedback to be more interpretable. Overall, most participants indicated that they would like to continue using the mRehab system at home.

**Conclusions:**

Assessing usability in the lived environment over a prolonged duration of time is essential to identify the match between the system and users’ needs and preferences. While mRehab was well accepted, further customization is desired for a better fit with the end users.

**Trial Registration:**

ClinicalTrials.gov NCT04363944; https://clinicaltrials.gov/ct2/show/NCT04363944

## Introduction

There are approximately 7 million survivors of stroke in the United States [1]. Up to 60% have residual impairments, which in turn could limit their performance of daily activities [2]. While individuals with stroke are commonly given written home programs when they are discharged from traditional therapies, adherence to written home programs is poor [3]. Qualitative analyses suggest low adherence is related to finding the exercises boring, receiving poor feedback during exercise performance, and uncertainty in how to perform the exercises [3,4]. Mobile health (mHealth) apps provide new options for long-term rehabilitation. In 2018, 91% of adults over the age of 65 years owned a cell phone. Smartphone ownership has increased from 11% in 2011 to 53% in 2018 [5]. As of December 2017, almost 325,000 mHealth apps had been created [6]. However, only a small number of mHealth apps has been specifically designed for people with disabilities, and an even smaller number of apps has undergone accessibility evaluation with people with disabilities [7,8]. Fully assessing usability is critical for the effective and efficient use of mHealth interventions. User feedback on mHealth interventions indicates not all mHealth devices are easy to use [9,10], and this has the potential to limit user adherence. A high dropout rate is one of the most significant barriers to mHealth adoption [11,12]. The average mHealth app costs US $425,000 to develop; however, 83% of mHealth app publishers report a discouraging number of fewer than 10,000 users who activate the app at least once a month [13]. By placing a more significant emphasis on usability for consumers and stakeholders, iterative improvements can reduce costs and enhance the long-term use and adoption of mHealth interventions [14-16]. Thorough usability testing is critical for the success of novel mHealth interventions.

**Figure 1:**
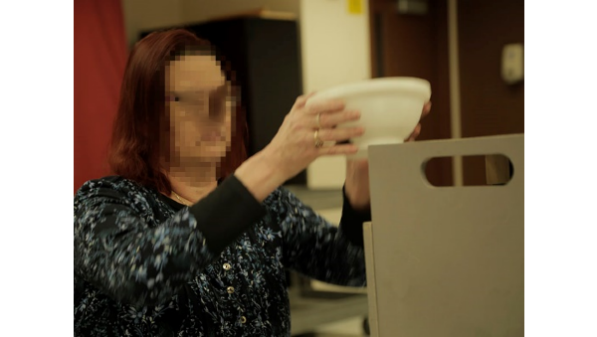
User transferring bowl with both hands.

**Figure 2:**
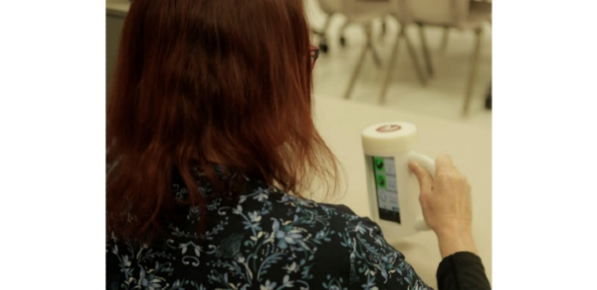
User seeing feedback on the smartphone screen inside the mug.

**Figure 3:**
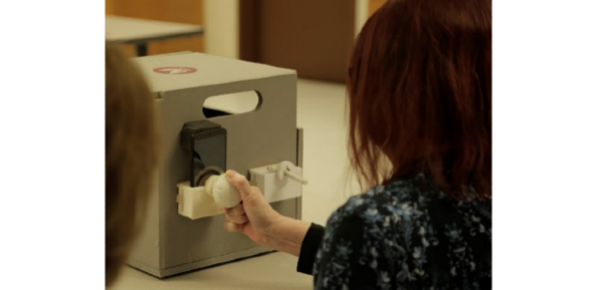
User turning doorknob with a smartphone in the holder and the key with a holder.

In previous work, a portable system for home rehabilitation, mRehab, was developed and reviewed by end users in a 1-day usability assessment and multiday assessment for consistency in measurement [17]. The system consists of a smartphone and 3D-printed objects in the shapes of household items (a bowl, mug, key, and doorknob; Figure 1). The 3D-printed objects were combined with the smartphone for 10 activities [17,18]. For example, the 3D-printed bowl was designed to hold the smartphone in a landscape orientation. The bowl depth was shallow and had a ridge along the top to allow the user to hold it with both hands (Figure 1). The mug was designed to hold the smartphone in an upright position. Security of the smartphone was ensured by using a screw-top lid on the mug (Figure 2). The mug had a cut-out window for the user to see the smartphone screen during activities. Both left-handed and right-handed mugs were designed. The key and doorknob had similar designs with a pocket holder for the smartphone and mechanical arm that swept across the screen as the object was turned (Figure 3). Two activities, Phone Number and Quick Tap, used the smartphone only and focused on fine motor movements. A wooden box was designed to hold all mRehab items and served as a mechanism to guide participants during horizontal and vertical transfer activities of the bowl or the mug.

**Figure 4:**
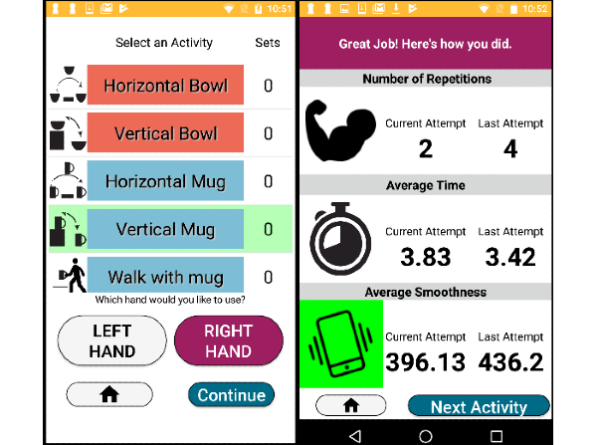
App interface: activity selection and feedback pages.

A Google Nexus 5 phone was used during all mRehab activities. We developed a mobile app that recorded movement-related data (duration and smoothness). This custom app allowed participants to select activities (Figure 4) and then record his or her performance on the activities. Once participants selected an activity, the app provided instructions to guide the user through the activity. A printed manual with instructions was also provided to each user [17]. Additionally, on completion of an activity, the app provided visual feedback in the form of performance scores on the number of repetitions completed, average time to complete a repetition, and average smoothness with which the repetition was completed (Figure 4). The app also provided an auditory readout of the scores. Different from existing technology-based rehabilitation tools, mRehab provides a set of realistic rehabiliaton activities mimicking activities of daily living (ADLs), utilizes a task-oriented approach that focuses on function, and is client-centered. A detailed description of each activity is found in a previous publication [17]. The app also provided performance feedback allowing the user to compare their current performance against their score from the previous session. When the participant’s performance (number of repetitions, average time, average smoothness or accuracy) improved over the previous session, the specific icon turned green (eg, average smoothness in Figure 4) and made a celebratory auditory tone to notify the participant they improved [17]. The user could also view a graph that plotted his or her scores from the prior 6 weeks. Previously, we reported on the usability assessment of the previous prototype of mRehab and modifications made that led to the current prototype. We also reported on the consistency of the app measurement for each activity using the current prototype [17].

In this work, the usability assessment of the system was conducted after a more robust usage of mRehab for 6 weeks at home by 11 individuals with stroke. The examination of usability, usefulness, and acceptance of mRehab holds importance beyond developing this system. Lessons learned about the form and function of mRehab have broad application to mHealth. The use of technology to support home rehabilitation is timely as recommendations to stay home during the COVID-19 pandemic are requiring modifications to health care delivery.

## Methods

### Research Design

We used a mixed-methods approach, which included quantitative surveys to evaluate long-term usability and perceived usefulness of mRehab, and evaluated the acceptance of the mRehab system. Semistructured interviews with participants were used to further elaborate on the strengths and weaknesses of the mRehab system to better understand the essential ingredients to develop a robust and user-friendly system. The study was approved by the University at Buffalo Institutional Review Board.

### Participants

We used a convenience sampling approach to recruit 11 individuals with stroke from the Western New York region who were (1) 18 years of age or older, (2) community dwelling, (3) an independent ambulator, and (4) at least 6 months post stroke. Participants were excluded if any of the following conditions interfered with their participation: (1) cognitive impairment indicated by score of 123 or lower on the Mattis Dementia Scale; (2) acute or chronic pain that would interfere with participation in the study (based upon participant’s self-evaluation); (3) severely limited range of motion or contractures of the shoulder, elbow, wrist, or hand that would interfere with participation in the study; (4) absence or severely impaired proprioception of the upper limb; (5) musculoskeletal or circulatory conditions affecting the upper limb; (6) severe spasticity; or (7) recent treatment (within 3 months) for spasticity including botulinum toxin injections or spasticity medications including intrathecal baclofen. Due to a limitation in the number of mRehab units, participants were recruited in 2 rounds: 5 in the first and 6 in the second. All participants provided written informed consent prior to initiating the study.

### Procedures

Participants completed 2 in-lab sessions prior to starting the home program. During these sessions, they completed a demographic questionnaire, clinical assessments including the 9-hole peg test and Wolf Motor Function Test, and assessment of hand grip strength and received, in total, 40-60 minutes of training on the mRehab system. In the lab, participants received instructions to select the activity on the mRehab app, insert the smartphone into each 3D-printed object, perform each activity, and interpret the feedback [17]. Each participant then proceeded to independently complete setting up the mRehab system and perform each activity for 3-5 repetitions to indicate that they were comfortable with setting up and completing the sessions independently. We also explained to the participants that the Quick Twist Mug activity was optional. This activity had lower measurement consistency than we wanted for recommendation in the home program [17], but for those participants willing to use the activity, long-term feedback on performing the activity was considered helpful in furthering the mRehab system. Participant requests for customization such as increasing the font size in the mRehab app for better readability were addressed. For the home program, an occupational therapist suggested that the participants perform 10 repetitions of each activity, 5 times per week as quickly and smoothly as possible. It was clarified that this was only a suggestion and that participants could choose to do more or fewer repetitions. Participants used mRehab at home for 6 weeks and were instructed to contact researchers if they encountered difficulties. After 6 weeks, participants returned to the lab and completed the clinical assessments, showing improved performance [18], and several structured questionnaires. Two questionnaires assessed their general perception towards exercise and technology, the Self-Efficacy for Exercise Scale and the Attitude toward Technology, respectively. The other questionnaires were specific to mRehab: (1) System Usability Scale (SUS); (2) mRehab Acceptance Questionnaire, based on the Technology Acceptance Model; and (3) Difficulty Rating Scale (DRS). Details of each instrument are included in the following sections. Each participant then engaged in a 1-hour retrospective interview conducted by a member of the research team to discuss their experience with using the mRehab system at home. The semistructured interview questions are summarized in Multimedia Appendix 1.

### Instruments

#### Hand Grip Strength Assessment

Hand grip strength assessment using a handheld dynamometer was conducted as part of the Wolf Motor Function Test [19,20]. Hand grip strength assessments were performed for the individuals’ affected and nonaffected sides to indicate the individual’s baseline motor ability [21].

#### Self-Efficacy for Exercise (SEE) Scale

On a scale of 1-10, participants indicated their self-efficacy related to exercising in general. Higher scores indicate that participants were more confident that they would complete the exercise when they were alone, stressed, depressed, etc [22].

#### Attitude Toward Technology

On a scale of 1-7, participants indicated their attitude toward the use of technology in general. Higher scores indicate an increased likelihood that the participant was enthusiastic about using new technology. These questions are based on the Technology Acceptance Model [23-25] and are summarized in Multimedia Appendix 2.

#### System Usability Scale (SUS)

The SUS has been previously used for assessing usability of mobile rehabilitation apps and systems [26,27]. The SUS consists of 10 questions, each rated on a 5-point Likert scale [28], to assess the participant’s satisfaction with the whole mRehab system. The SUS is a reliable and valid measure of the perceived usability of a system [29,30] and has been used with small sample sizes of 8-15 users [31,32]. The SUS was used to assess the participant’s satisfaction with the mRehab system.

#### mRehab Acceptance Questionnaire

The mRehab Acceptance Questionnaire was based on the original Technology Acceptance Model and the extended models [33-35]. The questions addressed the mRehab system as a whole and asked about the participant’s perception of the system usefulness and ease of use, learnability of the system, self-efficacy for mRehab usage, attitude toward mRehab, and behavioral intention to use the mRehab system in the future. The questions were modified from previous literature [23,36-40] and used a 7-point Strongly Disagree to Strongly Agree Likert-type scale. The questions are summarized in Multimedia Appendix 2.

#### Difficulty Rating Scale (DRS)

**Figure 5:**
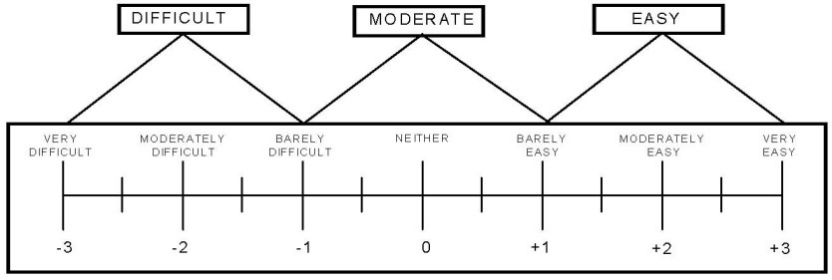
Ordinal scale on the Difficulty Rating Scale (DRS).

The DRS focused specifically on the hardware design of each of the 3D-printed objects (mug, bowl, key, and doorknob), and elicited participant opinions on their ease of use. Participants rated the ease of use on a scale ranging from Very Difficult to Very Easy (Figure 5).

#### Semistructured Interview

The interview questions elaborated on the usability of the system, including what they liked or disliked about the system components, activities that they benefitted from, and activities that were preferred. Based on the participant responses to the initial probes (see Multimedia Appendix 1), follow-up questions had participants elaborate on their use of the 3D-printed objects and their respective rehabilitation activities.

### Data Analysis

Demographic variables are descriptively summarized in Table 1. For the SUS, percentile ranks were calculated from participant ratings of their perceived usability [41]. Grades were assigned to percentile ratings from Grade A to D as recommended by Sauro in 2018 [41]. The assigned cut points for the grades were as follows: A+: 96-100; A: 90-95; A-: 85-89; B+: 80-84; B: 70-79; B-: 65-69; C+: 60-64; C: 41-59; C-: 35-40; and D: 15-34, with grade B- or better indicating acceptable usability and D indicating marginal acceptability. The average of the ratings was calculated for each participant for each subsection of the mRehab Acceptance Questionnaire. Then, the mean and SD were calculated for the mRehab Acceptance Questionnaire for each question across participants [25]. Pearson product moment correlation was used to evaluate the relationship between participants’ average number of repetitions performed and their ratings on the SUS and mHealth Acceptance Questionnaire. Use was quantified based on the average number of repetitions per activity over the 6 weeks. Changes in clinical assessments were also examined using the Wolf Motor Function Test and have been reported in another paper [18].

**Table 1:** Participant characteristics.

ID	Age (years)	Gender	Affected side	Reported dominant arm prior to stroke	Hand grip strength (lb)			Prior experience in using			SEE^a^ Scale (1-10 scale), mean		Attitude toward technology (1-7 scale), mean
					Affected side	Nonaffected side	Mobile phone		Smartphone				
S01	57	F^b^	R^c^	R	20	41.7	Y^d^		Y	6.4		2.3	
S02	54	F	L^e^	L	25	45	Y		Y	8.2		5.7	
S03	68	M^f^	R	R	30	80	Y		Y	10		4.7	
S04	61	F	R	R	28.3	41.7	Y		N^g^	6.8		3.3	
S05	78	F	L	R	28.3	51.7	Y		N	10		4.7	
S06	66	M	L	L	30	111.7	Y		Y	6.9		5	
S07	73	M	L	L	10	58.3	Y		N	3.6		3	
S08	61	M	L	R	61.7	73.3	Y		Y	6.9		3	
S09	62	F	R	R	5	40	Y		Y	6.4		3.3	
S10	67	M	R	R	60	60	Y		Y	8.9		2.3	
S11	76	M	R	R	45	48.3	Y		N	8.7		2.3	
Mean (SD)	65.7 (7.7)	N/A^h^	N/A	N/A	31.2 (18.1)	59.3 (21.8)	N/A		N/A	7.5 (1.9)		3.6 (1.2)	

^a^SEE: Self-Efficacy for Exercise.

^b^F: female.

^c^R: right.

^d^Y: yes.

^e^L: left.

^f^M: male.

^g^N: no.

^h^N/A: not applicable.

All interviews were audio recorded and transcribed verbatim by a professional transcription agency. The first author reviewed each transcript for accuracy. QSR’s NVivo 12 was then used to code themes within the transcripts. Thematic analysis was used to identify and extract themes, explain what each theme could mean, and determine links between themes. The first author and a research assistant independently coded the transcripts to identify primary and secondary themes from the interview transcripts. Both reviewers discussed their coding once per week over a 6-week coding period and reached mutual consensus in case of any disagreement about coding.

## Results

### Participant Demographics

The study sample included 11 individuals with stroke, with a mean age of 65.7 (SD 7.7) years and age range of 54-78 years, and 5 of 11 participants were female (46%; detailed in Table 1). On average, the participants were over 7 years poststroke. Of the 11 participants, 8 (73%) were right-side dominant prior to stroke, and 9 (82%) reported that their dominant side was the affected side poststroke. All participants had prior experience with using mobile phones, and most participants (7 out of 11) had prior experience with using a smartphone. On the Attitude Toward Technology, participants reported a mean score of 3.6 (1.2) on the 7-point Strongly Disagree to Strongly Agree scale. All but one participant indicated high self-efficacy for exercise, ranging between 6.4 to 10 in general.

### Participant Completion

All but one participant completed the 6-week in-home rehabilitation program. While the participant did not complete the in-home program, they did complete the postintervention interview and all the questionnaires. During the interview, the participant explained that she needed her caregiver to be present during the mRehab sessions. She had difficulty with setting up the mRehab activities and needed support. To better understand this participant’s experiences with mRehab, her ratings were included in all reported results.

### Issues With the mRehab System

During the in-home period, 6 participants (4 from the first group and 2 from the second group) contacted the research team with reports of breakage in the mRehab system. A majority of the participants in the first group experienced breakage of the doorknob (n=4) and the key (n=2). In case of breakage, the 3D-printed items were replaced within 1-2 days. Following the completion of group 1, we upgraded the 3D-printed items with larger infill to make the doorknobs and keys stronger to withstand repetitive use. In group 2, only 2 participants experienced doorknob breakage.

### Perceptions of the mRehab System

Table 2 includes individual-level perceptions of the mRehab system. The SUS scores indicate that all but one participant were satisfied with the usability of the mRehab system. Most participant ratings (10/11) ranged from the 67.5th to the 97.5th percentile, which were Grade B- or better. Participants (11/11) also provided favorable responses on the mRehab Acceptance Questionnaire (a 7-point scale), with a mean perceived usefulness of 5.7, mean perceived ease of use of 5.3, and mean self-efficacy for mRehab usage of 6.0. Also, mean ratings for participants’ attitudes toward mRehab was 6.3, and participants’ behavioral intention to use mRehab in the future was 5.3. Individual questions for each construct in the mRehab Acceptance Questionnaire have been summarized in Multimedia Appendix 2. For the question “Learning to operate the system was easy for me,” participants (11/11) provided a mean rating of 6.1. The average total repetitions of all activities combined per day from the mRehab app are also summarized in Table 2. The correlations between average number of repetitions per day and ratings on SUS, mRehab Acceptance Questionnaire, or DRS were small, and none reached an alpha of .05.

**Table 2:** Participant ratings on the System Usability Scale (SUS) and mRehab Acceptance Questionnaire and their performance with the mRehab system.

ID	SUS (1-10 scale)			Perceived usefulness (1-7 scale)		Perceived ease of use (1-7 scale), mean		Average repetitions in 6 weeks for all activities
	Percentile	Grade						
S01	17.5	D	5		1.2		N/A^a^	
S02	97.5	A+	7		6		189	
S03	87.5	A-	7		6.4		255.8	
S04	85	A-	6		4.4		256.8	
S05	65	B-	5		6.2		461.1	
S06	67.5	B-	6		5.8		62.3	
S07	80	B+	6		5.2		216	
S08	82.5	B+	4		4.6		132.7	
S09	80	B+	6		5.4		195.3	
S10	67.5	B-	5		6		106.2	
S11	95	A	6		5.8		461.5	

^a^N/A: not available because the participant did not complete the study.

Participant responses on the DRS indicated that the majority of participants found the mug and bowl easy to use. On the DRS, 7 participants found the mug easy to use, and 4 found it moderately easy to use; 8 participants found the bowl easy to use, and 3 found it moderately easy to use. However, more difficulty was reported with the ease of use of the key and the doorknob. For the doorknob, 3 participants reported it easy to use, 3 reported it as moderate, and 5 indicated it was difficult to use. For the key, 5 participants reported it easy to use, 3 reported it as moderate, and 3 indicated it was difficult to use.

### Themes

The discussion themes identified from the participant interviews are summarized in the following sections: usability of the mRehab system, usability of the performance-based feedback system, usefulness of mRehab activities, support needed with use of mRehab, and generalization to new activities of daily living. The frequency of participant responses reported in the qualitative results represents the number out of all 11 participants.

### Usability of the mRehab System

#### Hardware Design

Comments about the design of the 3D-printed objects were largely positive. Of the 11 participants, 9 liked the bowl, 8 found the mug “good,” and 5 liked the doorknob. Comments regarding the design of the doorknob included “an excellent design” and “it was easy to get ahold of it.” Regarding the key, 6 participants said that although the key size was bigger than a typical key, they preferred the bigger size for training. The current shape and size allowed a good grip on the key when turning. Some participants pointed out that they would prefer customization of the bowl and mug handle based on the participant’s hand size and potentially adding a textured grip on the handle. And 2 participants suggested using a latch or a handle-lever shaped doorknob in the future.

#### Hardware Functioning

**Figure 6:**
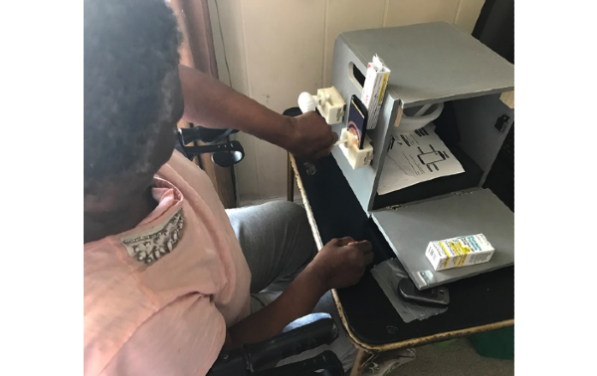
Participant using a pill box behind phone when engaging in Turn the Key activity.

When using the mRehab system at home, 8 participants reported leaving the system set up on a table. Participants thought that the bowl was easy to use during exercise. No difficulties were reported by participants on how to use the mug for the mRehab activities. Regarding using the mug, 5 participants stated that they found the mug easy to use and that the phone was easily accessible when inside the mug. Two participants reported repeated breakage of the doorknob, which led to lower average repetitions for the Turn Doorknob activity. The first 7 participants reported that the doorknob design prevented continuous pairing of the contact interface between the doorknob with the smartphone screen. Some of these participants reported being worried that this could lead to erroneous calculation of smoothness and therefore actively fixed the issue by either placing rolled up paper napkins or a pillbox behind the phone (Figure 6). Additionally, the research team made home visits to attach a piece of foam on the box that pushed the smartphone forward and minimized the space between the smartphone and doorknob, thereby fixing this issue. Since the design of the key was similar to that of the doorknob, there was a similar problem. For 8 participants, initially the app did not register the movement of the key on the phone screen. Again, using an object to push the phone forward toward the key worked well.

#### Software Design

All participants switched the phone off to preserve battery. Participants reported that the design of the app interface needed to be refined to allow them to make choices on the screen while the phone is in the key or doorknob holder. Two participants reported being pleased by the customizable nature of the app that allowed them to view larger fonts on the screen.

#### Software Functioning

Two participants reported being confused by the repetition count by the app when they engaged in activities. Participants thought that the app count was directive and they were expected to perform a repetition after the app had counted. The participants reported that they had forgotten that the app counted only after they had completed a repetition. Also, the app count had a brief time lag in counting, which some participants reported to be confusing.

### Usability of the Performance-Based Feedback System

#### Difficulties With the Feedback

Of the 11 participants, 5 participants stated that they did not understand the numbers on the feedback screen and that scores that went to 3 decimal places were not meaningful. One participant explained that they forgot the significance of the auditory celebratory sound and an icon turning green on the feedback screen:

I really didn’t know [laughs] what I was supposed to be doing–what improvement was. Each time I tried to do them. I was trying to do them as smoothly as I could, and then I was trying to do them all.

This participant also reported forgetting to look at the manual for a description of the feedback. Although the app was designed to allow participants to see the history of their performance as a line graph over the 6-week period, all participants who remembered the “History” tab (9/11) reported that the app crashed consistently when the history tab was opened. Two participants forgot that the app had a “History” tab and did not remember to look at the manual for more details.

#### Positives About the Feedback

Of the 11 participants, 9 participants said that they liked the green light and the auditory note of the feedback. One of these participants explained that she deliberately performed 2 sets of each activity everyday with at least one additional repetition in the second set. Performing one extra repetition compared to the previous set ensured that her feedback had at least one green icon for repetitions. One participant explained that the green icon let them identify the activities in which they were becoming “proficient.” Another participant said:

I liked it when it gave you stats like how well you did, the green light, saying, “Woo! Strong!” that you're getting stronger there and increasing the repetitions. I like the noises that it made.

One participant said that they tried to redo the activities to get a green icon.

#### Suggestions for Feedback

Several participants offered suggestions to improve the feedback system; 4 participants said that seeing or hearing the feedback in words could be helpful such as “Today you did faster than yesterday.” One participant explained that he would prefer to know what the app was measuring and how he could improve his performance. One participant pointed out that in the activity Walk with Mug, the phone made an initial spilling sound and then stopped. A continuous spilling sound would help.

Two participants said that they would like to see negative feedback. One participant’s caregiver explained that the negative feedback could motivate the participant to try another set. One participant requested to include an option to see best score since start. She said:

I did it a lot. It got lost. I couldn't tell you what my best score was.

### Usefulness of mRehab Activities

#### Beneficial Activities

Of the 11 participants, 10 participants reported that they benefitted in some way from one or several of the mRehab activities. Some participants selected more than one activity. Phone Number, Transfer Mug Vertically, and Slow Pour were reported as beneficial by 3 participants. One participant explained that the Slow Pour activity was beneficial for her because it resembled a real-life task. Another participant explained that the horizontal and vertical mug activities were beneficial for her and said, “I can feel it in my shoulder.” Phone Number and Quick Tap were reported as beneficial by 2 participants because they required fine motor skills and helped to improved hand-eye coordination. Quick Twist Mug and Transfer Bowl Vertically were not reported as beneficial by any of the participants. Further detail was not provided by 4 participants who reported benefitting from an activity.

#### Favorable Activity

One or more favorable activities were reported by 10 participants. The only activity not mentioned as a favorite was Turn Doorknob, and the activity mentioned the most, by 5 different participants, was the Transfer Mug Horizontally. The participants did not explain why they enjoyed the activities; they just stated that they liked certain activities more than others.

#### Nonbeneficial Activities

Eight participants reported not using the Quick Twist Mug activity at all. One of these participants explained that for Quick Twist Mug, the app needed her to quickly supinate and pronate her forearm, and her movement was not quick enough for the app to count the repetition. Walk with Mug and the Transfer Mug Vertically were chosen by 2 participants as nonbeneficial. Turn Doorknob, Turn Key, and Transfer Mug Horizontally were mentioned as nonbeneficial only once. Three participants said that some activities were not beneficial since they were too easy, or they were already able to perform the action with ease before starting the mRehab program.

#### Nonfavorable Activities

The 4 nonfavorable activities were Slow Pour, Quick Tap, Sip from Mug, and Walk with Mug. Slow Pour was identified as the least favorable activity by 4 participants; 2 of these participants explained they did not like Slow Pour because it forced them to move slowly and they wanted to move faster.

### Support Needed to Use mRehab

Four participants indicated that their caregiver helped when using the mRehab system; 3 participants reported needing help with navigating the app, and 1 of the participants felt they could have used the app independently, but defaulted use of the app to the caregiver because they were more familiar with smartphones. All 4 participants needed physical support with setting up the mRehab activity components. This ranged from assistance with lifting the box to physical assistance with setting up activities. One of the participants indicated that going through all the mRehab activities would take 40-45 minutes and that it was difficult to find free time where their caregiver was available to sit down and help for the entire time.

### Generalization to New Daily Life Activities

Nine participants reported initiating a new skill following use of the mRehab system, and 9 participants described an increase in control and use of their affected upper extremity or hand post-mRehab activities. Various ADL performances were brought up by participants: pouring laundry detergent, washing dishes, drying dishes, wiping off countertops, stabilizing with the affected hand, donning socks, opening doorknobs, taking clothes out of dryer, and gripping objects more often. Two participants reported an increase in dexterity of their affected hand post-mRehab activities. Four participants said they were more conscious of using the affected hand during ADLs to continue practicing using it, even outside mRehab activities. Two participants said they did not start doing any new activities, and 1 said it was because they were still experiencing residual pain in their affected hand from their stroke.

## Discussion

All participants, except for 1 participant, completed the 6-week study. Overall, participants indicated that they liked using the mRehab system at home and that they benefitted from its use. High percentile ranks on the SUS and high mean ratings on the mRehab Acceptance Questionnaire indicate that the mRehab system was useful as a remote home program and that participants were satisfied with the usability of the system. Although it is possible that individuals who were comfortable with the use of technology volunteered to participate in this study, low scores on the Attitude Toward Technology indicate that the recruited participants were, in general, typically hesitant to try out new technology.

For this study, the inclusion and exclusion criteria were created to ensure that individuals had sufficient function to interact with the system. The criteria, however, did not create a ceiling for the participants. The degree of deficits for individuals varied in the study [18]. By virtue of participants requesting to be in the study, it indicates that they perceive deficits that they would like to improve with a home program. Mild stroke is not uncommon [42], and providing avenues for motor improvement is also important for this group.

The convergence of the qualitative and quantitative data supports the strengths of using a mixed-methods design for capturing a holistic picture for system usability [43]. Participants’ ease-of-use ratings and their interview responses indicate that the usability of the mRehab system was high. Participants who described that the bowl and doorknob were easy to use in their interviews also rated them to be +1.5 or higher on the DRS, indicating that they were easy to use. Similarly, participants who described that the design of the 3D-printed key needed to be customized or modified for ease of use rated the key to be moderate to difficult to use on the DRS.

Evaluation of usability over a longer period of time is critical because it portrays the challenges of using a system in day-to-day life while accounting for breakdowns and failures from repeated use. Participants experienced some breakage of the 3D-printed items resulting from repeated and prolonged use. Although the 3D items in the mRehab system had undergone usability testing and were modified based on participant feedback [17], extended use uncovered aspects of the mRehab system that can be improved and expanded in future developments. Participants emphasized the need for customizing the daily use objects in the mRehab system. Also, interviews with the participants revealed technical problems with the “History” tab, which was a newly added feature that was not pilot tested in previous iterations. Despite these issues, the majority of participants provided a grade of A- or better for mRehab on the SUS. Scores that are 68th percentile or higher on the SUS suggests future use of the system [16,28,29]. Both the perceived usefulness and perceived ease-of-use scores suggested the participants were satisfied and were accepting of the mRehab system. The Technology Acceptance Model posits that perceived usefulness and perceived ease of use are 2 main factors that predict actual use of the technology by the user and influence acceptance [23,44].

Although participants reported quickly learning to use the system in the mRehab Acceptance Questionnaire, the interviews revealed that they did not have a full understanding of the app interface or the feedback system. Over the 6-week period, participants had forgotten what the scores (numbers) meant, what the visual feedback (green light) was, and what the celebratory auditory note meant. These behaviors indicate that 40-60 minutes of training was not adequate for the participants to use the system to its fullest capacity in a remote setting. Relatedly, hospital-based research suggests transition planning and early training prior to discharge from hospital are important to facilitate carry over of skills to remote rehabilitation and promote self-management [45]. All participants had received a manual explaining the meaning and significance of each activity and the app interface; however, the participants reported either forgetting about the manual or not taking it out of the box. This indicates that the participants relied on the app to guide them through the entire exercise session. Better understanding how to support individuals in long-term home programs through in-person training and app design are important considerations for design and implementation of mHealth.

The long-term use of mRehab combined with multiple assessments of usability testing start to illuminate the individual’s preference for activities that are just right and are neither too easy nor too difficult. Participants’ preferences for the just-right amount of challenge have been demonstrated in previous literature [46,47]. Participants explained that they did not benefit from activities that were too easy. Conversely, several participants stopped using the Quick Twist Mug activity because it was too challenging. Also, with the Slow Pour activity, participants listed it as “not a favorite,” but reported they did the activity and found it beneficial. Taken together, it suggests that feeling appropriately challenged and benefiting from an activity are important aspects to consider in designing rehabilitation systems.

This was a small-scale, mixed-methods study to explore the feasibility of using mHealth relatively independently for upper limb rehabilitation by individuals with stroke. This sample size may not have allowed us to identify all the possible accessibility features needed by people with disabilities, but the in-depth conversations with these study participants enabled us to identify several major accessibility features desired by individuals with stroke. Additionally, despite immediate replacements, the breakage of some of the 3D-printed items may have caused negative perceptions about the mRehab system. However, the participants provided an overall positive usability rating for the mRehab system. The first group of participants experienced a higher incidence of breakage than the second group. Although our plan did not entail using an iterative approach within this study, the first group’s home use of the 3D-printed items allowed us to modify the 3D-printed objects for the second group. The benefits of extended use of a device prior to usability testing are well illustrated in this study.

During screening, participants were included if they indicated in their self-assessment that pain would not interfere with their participation. Experiencing pain is a common clinical consequence after stroke [48], and nearly 70% of poststroke patients experience pain on a daily basis [49]. Postintervention, 2 participants reported not engaging in new activities, fearing pain. The usability assessments in this study did not fully evaluate if mRehab activities resulted in pain. At the start of the study, participants were instructed to stop mRehab activities if they experienced increased pain and to contact the research team. No participant contacted the research team with complaints of pain. Perceived fear of pain when performing a new activity may also impact the participant’s willingness to engage in new activities. In previous studies, participants reported planning daily activities with their nonaffected side due to fear of injury to their affected arm [50,51]. In future studies, a pain scale on the mobile app that records reports of pain and assessing fear of pain with movement will help clarify how pain and the fear of pain impact outcomes. This line of study is important in better understanding how training in rehabilitation programs may transfer to movement outside of the rehabilitation program.

Assessing usability and usefulness of mHealth interventions is critical to incorporate user opinions and customize the intervention to the users’ needs and preferences. It is not common for end users to evaluate their exercises [52], let alone assess long-term usability in the user’s lived environment. Findings from this study indicated users’ preferences for (1) realistic design of the 3D-printed objects, (2) activities resembling daily living tasks, (3) customizable nature of the app, (4) being adequately challenged by the activities, and (5) performance-based objective auditory and visual feedback.

## Appendix

Multimedia Appendix 1 Format of the semi-structured interview.

Multimedia Appendix 2 Questions based on Technology Acceptance Model.

## Abbreviations

ADLs: activities of daily living
DRS: Difficulty Rating Scale
mHealth: mobile health
SEE: Self-Efficacy for Exercise
SUS: System Usability Scale
