# Supplementary material for: Usability, Usefulness, and Acceptance of a Novel, Portable Rehabilitation System (mRehab) Using Smartphone and 3D Printing Technology: Mixed Methods Study
Source: JMIR Hum Factors. 2021 Mar 22;8(1):e21312. doi: 10.2196/21312 (PMC8080267; doi:10.2196/21312)
Supplement: Multimedia Appendix 1 [file humanfactors_v8i1e21312_app1.docx]

Multimedia Appendix 1. Format of the semi-structured interview

| **Regarding the hardware (3D printed items)** |
| --- |
| 1. What did you like best about the mug? Why? 2. What did you like least about the mug? Why? 3. How *difficult or easy* was it to use the mug? |
| 1. What did you like best about the bowl? Why? 2. What did you like least about the bowl? Why? 3. How *difficult or easy* was it to use the bowl? |
| 1. What did you like best about the doorknob? Why? 2. What did you like least about the doorknob? Why? 3. How *difficult or easy* was it to use the doorknob? |
| 1. What did you like best about the key? Why? 2. What did you like least about the key? Why? 3. How *difficult or easy* was it to use the key? |
| 1. What additional components would you like to see? 2. Do you have suggestions for modifying the existing 3D objects or the app? |
| **Regarding the software (app)** |
| 1. What did you like best about the app? Why? 2. What did you like least about the app? Why? 3. How *difficult or easy* was it to use the app? |
| **Regarding the feedback system** |
| 1. What did you like most about the feedback that you received? 2. What did you like least about the feedback that you received? 3. Do you have any suggestions for additions or modifications to the feedback system? |
| **Regarding the questions on the questionnaires** |
| - Please feel to expand or elaborate on any of the questions from the System Usability Scale or the mRehab Acceptance Questionnaire. |
| **Regarding the mRehab activities** |
| 1. Which activity was your favorite? Why? 2. Which activity was your least favorite? Why? 3. From which activity did you benefit the most? Why? 4. From which activity did you not benefit at all? Why? |
| **In general** |
| - Since you started using the mRehab system at home, have you tried to do any daily activities using your affected side that you previously had stopped doing? - If yes, what are they? |
