# Supplementary material for: Usability, Usefulness, and Acceptance of a Novel, Portable Rehabilitation System (mRehab) Using Smartphone and 3D Printing Technology: Mixed Methods Study
Source: JMIR Hum Factors. 2021 Mar 22;8(1):e21312. doi: 10.2196/21312 (PMC8080267; doi:10.2196/21312)
Supplement: Multimedia Appendix 2 [file humanfactors_v8i1e21312_app2.docx]

Multimedia Appendix 2. Questions based on Technology Acceptance Model

| **Construct** | **Questions (7-point scale) Strongly Disagree to Strongly Agree** | **Mean (SD)** |
| --- | --- | --- |
| **Attitude toward Technology in general** | | |
| Attitude toward technology | - If I heard about a new technology, I would look for ways to experiment with it | 4.1 (1.3) |
|  | - Among my peers, I am usually the first to try out new technology | 2.7 (1.6) |
|  | - In general, I am not hesitant to try out new technology | 4.0 (1.6) |
| **mRehab Acceptance Questionnaire** | | |
| Perceived usefulness | - Using this system would improve my performance | 5.7 (0.9) |
| Perceived ease of use | - I feel that using this system was easy for me | 6.1 (1.8) |
|  | - I feel that my interaction with the system was clear and understandable | 4.6 (1.5) |
|  | - Interaction with the system does not require a lot of mental effort | 4.5 (2.4) |
|  | - Learning to operate the system was easy for me | 6.1 (0.9) |
|  | - I found it easy to get the system to do what I want it to do | 4.6 (1.9) |
| Attitude toward usage | - I believe it is good idea to use the smartphone-based system | 6.1 (0.9) |
|  | - I like the idea of using the smartphone-based system | 6.4 (0.9) |
|  | - Using the smartphone-based system would be a pleasant experience | 6.3 (1.0) |
| Behavioral Intention | - If I have continued access to the system, I intend to use it | 5.6 (1.9) |
|  | - If I have continued access to the system, I want to use it as much as possible | 5.0 (2.3) |
| Self-efficacy | - I feel confident about using the smartphone-based system | 6.0 (1.8) |
|  | - I believe that I have the necessary skills for using the smartphone-based system | 5.8 (2.0) |
